# Supplementary material for: Fetal Bowel Abnormalities Suspected by Ultrasonography in Microvillus Inclusion Disease: Prevalence and Clinical Significance
Source: J Clin Med. 2022 Jul 26;11(15):4331. doi: 10.3390/jcm11154331 (PMC9332086; doi:10.3390/jcm11154331)
Supplement: Supplementary file 1 [file jcm-11-04331-s001.zip › jcm-1723661-supplementary.pdf]

Table S1. Cases of MVID with reported prenatal bowel abnormalities suspected by ultrasonography (A), without reported bowel abnormalities suspected by ultrasonography (B), and with reported uneventfull pregnancy or prenatal course (C)

A) CASES OF MVID WITH REPORTED PRENATAL BOWEL ABNORMAILITIES SUSPECTED BY ULTRASONOGRAPHY (n=28)

year	PMID / reference	Sex	Consanguini	Gestation	Birth weig	birth weig	Onset of c	Stool outp	Na+ (norm	Cl- (norma	K+ (norma	Feces osm	Feces pH	Dead, age	Ultrasonography report	time of ult	Mutation details
1991	1660676	m	n.r.	AT	3530	n/a	1	150	103	89	19	n.r.	n.r.	alive, nr	polyhydramnios	n.r.	before 2008
1991	1660676	m	n.r.	37	3300	79.66	1	150	122	102	19.4	n.r.	n.r.	alive, nr	polyhydramnios	n.r.	before 2008
1997	9323563	m	yes	33	2950	98.57	1	n.r.	99	n.r.	12	240	n.r.	dead, 7 m	polyhydramnios	n.r.	before 2008
2000	10941971	f	yes	34	2100	99.66	6	300-400	n.r.	n.r.	n.r.	n.r.	n.r.	alive, >24	bowel dilation	30	before 2008
2001	11251929	m	n.r.	n.r.	3720	n/a	3	n.r.	n.r.	n.r.	n.r.	n.r.	n.r.	alive, nr	polyhydramnios (AFI 23.5 cm), m	35	before 2008
2002	11883547	f	n.r.	AT	n.r.	n/a	21	n.r.	n.r.	n.r.	n.r.	n.r.	n.r.	alive, > 60	echogenic bowel	16	before 2008
2012	22318102	f	no	AT	2320	n/a	3	n.r.	35	21	n.r.	n.r.	n.r.	dead, 1 m	polyhydramnios, dilated bowel l	35	n.r.
2012	22197941	f	n.r.	37	2900	59.46	1	100	78	42	40	11	n.r.	alive, 4 d	polyhydramnios, bowel dilatatio	35	MYO5B mutations (unspecified).
2013	23525737	m	no	AT	n.r.	n/a	6	150	149	130	5	n.r.	n.r.	alive, >14	polyhydramnios	n.r.	n.r.
2013	23354788	f	no	35	2330	50.15	1	n.r.	85	78	22	n.r.	n.r.	dead, 7 m	large intestine dilation (18 mm)	3rd TM	MYO5B mutations (unspecified).
2013	25452882	f	n.r.	34	1975	37.34	12	>400	77	48	45	46	>6	dead, 1 m	polyhydramnios, NEC	n.r.	n.r.
2013	21199752	m	no	36	3355	92.46	1	n.r.	n.r.	n.r.	n.r.	n.r.	n.r.	dead, 6 m	polyhydramnios, multiple genera	29 and 36	MYO5B mutations: p.Q149* (pat), p.Q341* (mat)
2014	25547668	f	no	36	2555	48.80	3	150-200	106	n.r.	n.r.	n.r.	n.r.	dead, 16 n	bowel dilatation	n.r.	n.r.
2014	25547668	f	no	35	2320	49.13	3	150-200	113	n.r.	n.r.	n.r.	n.r.	dead, 29 n	bowel dilatation	n.r.	MYO5B mutations: p.1064*, p.Q1172*
2014	25547668 and 23608388	m	no	39	3680	77.42	4	200	104	n.r.	n.r.	n.r.	n.r.	alive, nr	bowel dilatation	n.r.	MYO5B mutations: p.Q149*, p.R1795*
2014	25111220	m	no	AT	3570	n/a	42	n.r.	n.r.	n.r.	n.r.	n.r.	n.r.	alive, 12 n	polyhydramnios, echogenic bow	34	MYO5B mutations: p.Ile408Phe, p.Leu528Phe
2017	27933652	f	n.r.	34	2100	50.76	3	n.r.	n.r.	n.r.	n.r.	n.r.	n.r.	dead, 3 m	polyhydramnios, non-obstructive	33	n.r.
2018	30564347	m	no	37	3280	78.44	10	n.r.	94	78	16	268	n.r.	dead, 7 m	polyhydramnios	n.r.	MYO5B mutations: p.Arg900Serfs*4, p.N456S
2019	30909251	m	yes	n.r.	n.r.	n/a	6	n.r.	n.r.	n.r.	n.r.	n.r.	n.r.	alive, nr	polyhydramnios, pre-eclampsia,	n.r.	STX3 mutations, p.Val122fs (hom)
2020	DOI: 10.1055/s-0040-1716401	f	yes	34		n/a	7	n.r.	n.r.	n.r.	n.r.	n.r.	n.r.	n.r.	polyhydramnios, MSAF	n.r.	STX3 mutations (hom)
2021	33544913	f	no	AT	n.r.	n/a	2	n.r.	n.r.	n.r.	n.r.	n.r.	n.r.	n.r.	dilated bowel loops	n.r.	MYO5B mutations: c.3277-2A>G (splice); deletion exon 24-27; = aa 1044-1201)
2021	http://dx.doi.org/10.1136/archdischild-201	m	yes	36	2960	73.31	2	350	n.r.	n.r.	n.r.	n.r.	n.r.	dead, 1 m	polyhydramnios, dilated bowel l	n.r.	MYO5B mutations: p.Gln1467* (hom)
2021	DOI: 10.15406/ogij.2021.12.00589	f	no	40	3420	58.99	3	n.r.	122	101	3.8	240	n.r.	dead, nr	polyhydramnios (AFI 15 cm), sigr	36	MYO5B mutations: c.1355-1363dup (pat), p.Ile488* (mat)
2021	33525641	f	yes	n.r.	n.r.	n/a	1	n.r.	n.r.	n.r.	n.r.	n.r.	n.r.	dead, <12	polyhydramnios. Patient id: 16D	n.r.	MYO5B mutations (unspecified)
2021	33663362	m	n.r.	36	2960	73.31	n.r.	n.r.	n.r.	n.r.	n.r.	n.r.	n.r.	alive, >12	polyhydramnios	n.r.	n.r.
2022	34816459	f	n.r.	37	3050	72.00	25	300	n.r.	n.r.	n.r.	n.r.	n.r.	dead, 1.5	polyhydramnios (AFV >91), dilate	35	MYO5B mutations: p.R219H (mat), p.Q536* (pat)
2022	34816459	m	n.r.	34	n.r.	n/a	5	n.r.	n.r.	n.r.	n.r.	n.r.	n.r.	dead, 3 m	polyhydramnios (AFV >70), dilate	34	MYO5B mutations: p. Trp874* (pat), exon11-12 del (mat) (= aa 441-515)
2022	35038185	f	n.r.	32	3420		n.r.	n.r.	n.r.	n.r.	n.r.	n.r.	n.r.	dead, 5 m	polyhydramnios and bowel dilati	n.r.	two MYO5B mutations (unspecified)

B) CASES OF MVID WITHOUT REPORTED PRENATAL BOWEL ABNORMAILITIES SUSPECTED BY ULTRASONOGRAPHY (n=19)

year	PMID / reference	Sex	Consanguini	Gestation	Birth weight (grams)		Onset of c	Stool outp	Na+	Cl-	K+	Feces osm	Feces pH	Dead/Aliv	Ultrasonography report	time of ult	Mutation details
1989	2759484	f	no	n.r.	2300	n/a	3	n.r.	100	82	29	n.r.	n.r.	n.r.	no polyhydramnios	n.r.	before 2008
1998	9740207	m	yes	AT	3900	n/a	8	175	105	74	12.1	281	8	dead, nr	no polyhydramnios	n.r.	before 2008
1994	7959671	m	yes	AT	4200	n/a	14	n.r.	n.r.	n.r.	n.r.	n.r.	n.r.	dead, 5 m	no polyhydramnios	n.r.	before 2008
1994	7959671	m	no	AT	3800	n/a	7	n.r.	95	n.r.	n.r.	n.r.	n.r.	alive, >57	no polyhydramnios	n.r.	before 2008
1994	8067796	m	yes	38	3325	65.39	1	150	58	36	15	309	n.r.	dead, 5 da	no polyhydramnios	n.r.	before 2008
1998	9822319	m	no	36	3090	81.64	1	>100	>100	119	111	14	5.5-7.5	alive, nr	no polyhydramnios	n.r.	before 2008
2001	11414303	m	yes	36	2700	50.98	2	n.r.	>100	60	18	n.r.	n.r.	alive, nr	no polyhydramnios	n.r.	before 2008
2001	11173328	m	no	36	2740	54.78	9	>250	65	73	25	n.r.	n.r.	dead, 17 n	no polyhydramnios	n.r.	before 2008
2008	11173328	m	no	34	2450	73.00	8	n.r.	112	113	21.6	292	n.r.	alive, nr	no polyhydramnios	n.r.	before 2008
2008	18277898	m	no	34	2450		8		112	113	21.6	292		alive, nr	no polyhydramnios	n/a	before 2008
2011	22030065	n/a	n.r.	23	n/a	n/a	n/a	n/a	n/a	n/a	n/a	n/a	n/a	n/a	no bowel dilation, no polyhydran	**<20 (!)**	MYO5B mutations: p.Q149* (pat), p.Q341* (mat)
2014	25111220	f	no	36	2950	81.08	2	220	n.r.	n.r.	n.r.	n.r.	n.r.	alive, 19 y	no antenatal ultrasound abnorm	n.r.	MYO5B mutations: p.Leu1055dup, p.Tyr755Glyfs*9
2014	25111220	m	no	AT	3290	n/a	7	n.r.	n.r.	n.r.	n.r.	n.r.	n.r.	alive, 24 n	no antenatal ultrasound abnorm	n.r.	MYO5B mutations: p.Ile408Phe, p.Leu528Phe
2014	25111220	m	no	AT	3360	n/a	5	160	n.r.	n.r.	n.r.	n.r.	n.r.	alive, 12 y	no antenatal ultrasound abnorm	n.r.	no MYO5B mutations found
2014	25111220	n.r.	n.r.	n.r.	n.r.	n/a	n.r.	n.r.	n.r.	n.r.	n.r.	n.r.	n.r.	alive, 13 y	no antenatal ultrasound abnorm	n.r.	no MYO5B mutations found/ possibly STX3 because of blindness
2014	25111220	m	no	31	1645	n/a	420	n.r.	n.r.	n.r.	n.r.	n.r.	n.r.	alive, 24 y	no antenatal ultrasound abnorm	n.r.	MYO5B mutations: p.Arg219His, p.Leu1343Pro
2014	25111220	m	no	n.r.	n.r.	n/a	60	n.r.	n.r.	n.r.	n.r.	n.r.	n.r.	alive, 28 y	no antenatal ultrasound abnorm	n.r.	MYO5B mutations: p.Phe450Leufs*30, p.Leu1055dup
2014	25111220	f	no	n.r.	n.r.	n/a	60	n.r.	15	n.r.	n.r.	n.r.	n.r.	alive, 11 y	no antenatal ultrasound abnorm	n.r.	MYO5B mutations: p.Phe450Leufs*30, p.Leu1055dup
2019	31559144	f	yes	34	2445	80.70	1	n.r.	137	98	n.r.	30	n.r.	alive, nr	no polyhydramnios (but large am	n.r.	MYO5B mutations: p.Ile488Leufs*93 (hom)

C) CASES OF MVID WITH REPORTED UNEVENTFUL PREGNANCY (n=25)

year	PMID / reference	Sex	Consanguini	Gestation	Birth weight (grams)		Onset of c	Stool outp	Na+	Cl-	K+	Feces osm	Feces pH	Dead/Aliv	Ultrasonography report	time of ult	Mutation details
1985	3977385	f	n.r.	37	2500		2	n.r.	91	n.r.	n.r.	n.r.	n.r.	dead, 6 m	n/a., normal pregnancy		before 2008
1985	3977385	f	n.r.	34	2200		4	n.r.	93	n.r.	n.r.	n.r.	n.r.	dead, 6 m	n/a., uneventful pregnancy		before 2008
1994	8067796	m	yes	35	2880		2	200	n.r.	n.r.	n.r.	n.r.	n.r.	n.r.	n/a., apparently normal pregnan	n.r.	before 2008
1994	7959671	f	yes	n.r.	4100	n/a	14	60	91	n.r.	n.r.	n.r.	n.r.	dead, 39 n	n/a., normal pregancy	n.r.	before 2008
1996	8732907	m	no	AT	3350	n/a	7	n.r.	n.r.	n.r.	n.r.	n.r.	n.r.	alive	n/a., normal pregnancy and uncomplicated		before 2008
1997	9364305	f	n.r.	36	n.r.	n/a	n.r.	15-200	n.r.	n.r.	n.r.	n.r.	n.r.	alive	n/a., uneventful pregnancy		before 2008
1997	9364305	m	n.r.	39	n.r.	n/a	3	n.r.	n.r.	n.r.	n.r.	n.r.	n.r.	alive	n/a., uncomplicated pregnancy		before 2008
1999	10484813	F	n.r.	35	2922		2	n.r.	n.r.	n.r.	n.r.	n.r.	n.r.	dead, 7 m	n/a., uneventful prenatal course		before 2008

1999	10484813	M	n.r.	34	2558		2	n.r.	n.r.	n.r.	n.r.	n.r.	n.r.	dead, 4 m	n/a., uneventful prenatal course	before 2008
1999	9880458	m	yes	39	3500		6	95	110	85	7	n.r.	n.r.	alive	n/a., uneventful prenatal course	before 2008
1999	9880458	m	yes	38	2600		6	135-165	81	44	2	n.r.	n.r.	alive	n/a., uneventful prenatal course	before 2008
1999	9880458	m	yes	36	3300		6	125-175	115	96	5	n.r.	n.r.	alive	n/a., uneventful prenatal course	before 2008
1999	9880458	f	yes	39	3160		4	150-200	107	84	18	n.r.	n.r.	alive	n/a., uneventful prenatal course	before 2008
1999	9880458	f	yes	37	2700		4	100	6	n.r.	27	n.r.	n.r.	dead, 36 n	n/a., uneventful prenatal course	before 2008
2001	11783915	m	yes	n.r.	n.r.	n/a	6	100-130	95	95	30	270	9.0	alive	n/a., normal pregnancy, labour and delivery	before 2008
2011	21968248	m	no	AT	2734	n/a	4	n.r.	84	68	13	n.r.	n.r.	alive	n/a., uneventful pregnancy	n.r.
2014	10.4236/ojpathology.2014.42010	m	yes	38	n.r.	n/a	2	n.r.	n.r.	n.r.	n.r.	n.r.	n.r.	dead, 2 m	n/a., no complications during pregnancy	n.r.
2014	10.4236/ojpathology.2014.42010	m	yes	34	n.r.	n/a	2	n.r.	n.r.	n.r.	n.r.	n.r.	n.r.	n.r.	n/a., no complications during pregnancy	n.r.
2014	25635218	f	no	36	2800		n.r.	120	83	n.r.	<50	n.r.	n.r.	dead, 9 m	n/a., uneventful pregnancy	n.r.
2016	27575604	f	yes	36	3145		3	n.r.	97	70	28		6.0	dead, mo1	n/a., uncomplicated pregnancy	MYO5B mutations: c.1323-2A>G (IVS10-2A) (hom)
2017	28842815	m	n.r.	38	3500		3	n.r.	78	64	7.3		7.0	dead, 1 m	n/a., normal antenatal scans	n.r.
2017	29546954	m	yes	36	n.r.		10	190	120	67	n.r.	n.r.	n.r.	alive	n/a., no pathological histories during pregna	n.r.
2017	28707991	m	yes	AT	3300	n/a	4	n.r.	n.r.	n.r.	n.r.	n.r.	n.r.	alive	n/a., uneventful pregnancy	n.r.
2017	29282386	m	no	AT	n.r.	n/a	3	n.r.	n.r.	n.r.	n.r.	n.r.	n.r.	n.r.	n/a., uneventful pregnancy	STX3 mutations (p.Arg247*) (hom)
2019	31049800	m	n.r.	n.r.	n.r.	n/a	n.r.	n.r.	n.r.	n.r.	n.r.	n.r.	n.r.	n.r.	n/a., no significant antenatal manifestation	n.r.

LEGEND

n.r.: not reported
n/a: not applicable
AT: at term
f: female
mo: months
PMID: PubMed identifier code
MSAF: meconium-stained amniotic fluid
AFI: amniotic fluid index
AFV: amniotic fluid volume
MVID: microvillus inclusion disease
hom: homozygous
pat: paternal
mat: maternal
